# 3D-Ultrastructure, Functions and Stress Responses of Gastropod (*Biomphalaria glabrata*) Rhogocytes

**DOI:** 10.1371/journal.pone.0101078

**Published:** 2014-06-27

**Authors:** Maria Kokkinopoulou, M. Alptekin Güler, Bernhard Lieb, Mike Barbeck, Shahram Ghanaati, Jürgen Markl

**Affiliations:** 1 Institute of Zoology, Johannes Gutenberg University, Mainz, Germany; 2 Institute of Pathology, University Medical Center of the Johannes Gutenberg University, Mainz, Germany; University of Copenhagen, Denmark

## Abstract

Rhogocytes are pore cells scattered among the connective tissue of different body parts of gastropods and other molluscs, with great variation in their number, shape and size. They are enveloped by a lamina of extracellular matrix. Their most characteristic feature is the “slit apparatus”, local invaginations of the plasma membrane bridged by cytoplasmic bars, forming slits of *ca.* 20 nm width. A slit diaphragm creates a molecular sieve with permeation holes of 20×20 nm. In blue-blooded gastropods, rhogocytes synthesize and secrete the respiratory protein hemocyanin, and it has been proposed–though not proven–that in the rare red-blooded snail species they might synthesize and secrete the hemoglobin. However, the cellular secretion pathway for respiratory proteins, and the functional role(s) of the enigmatic rhogocyte slit apparatus are still unclear. Additional functions for rhogocytes have been proposed, notably a role in protein uptake and degradation, and in heavy metal detoxification. Here we provide new structural and functional information on the rhogocytes of the red-blooded freshwater snail *Biomphalaria glabrata*. By *in situ* hybridization of mantle tissues, we prove that rhogocytes indeed synthesize hemoglobin. By electron tomography, the first three dimensional (3D) reconstructions of the slit apparatus are provided, showing detail of highly dense material in the cytoplasmic bars close to the slits. By immunogold labelling, we collected evidence that a major component of this material is actin. By genome databank mining, the complete sequence of a *B. glabrata* nephrin was obtained, and localized to the rhogocytes by immunofluorescence microscopy. The presence of both proteins fit the ultrastructure-based hypothesis that rhogocytes are related to mammalian podocytes and insect nephrocytes. Reactions of the rhogocytes to deprivation of food and cadmium toxification are also documented, and a possible secretion pathway of newly synthesized respiratory proteins through the slit apparatus is discussed.

## Introduction

Rhogocytes are prominent cells embedded in the connective tissue or floating in the hemolymph of gastropods and other molluscan taxa [1]. They are completely surrounded by a lamina of extracellular matrix that suggests a mesenchymal origin of this cell type. Their plasma membrane shows many invaginations that usually appear to be empty but occasionally contain granular material. More remarkably, these extracellular lacunae (also referred to as “subsurface cisternae”) are covered by finger-like cytoplasmic bars separated by distinct slits of 20 nm width. This peculiar slit apparatus (or “slit complex”) resembles a gully grate, with the difference that the slits are bridged by a thin diaphragm [2], [3]. The term rhogocyte (proposed by Fioroni *et al.* [4]) highlights this characteristic structure, because the Greek word *rhogos* means “slit”. Various other names have been given to this cell type, for example pore cells, Leydig cells, cellule nucale, Blasenzellen or brown cells (for review, see [1]). Morphologically, rhogocytes resemble the renal podocytes of mammals and insect nephrocytes [1]. These similarities, notably the dimensions of the slits, the slit diaphragm and the enveloping lamina of extracellular matrix, suggest a common developmental origin of rhogocytes, podocytes and neprocytes [1], [5], [6]. However, to-date this has only been proved for podocytes and nephrocytes by the identification of common slit diaphragm proteins [7].

Rhogocytes vary in size from 2 to 30 µm and can be round, elongated or irregularly shaped. The most prominent intracellular structures are a well-developed endoplasmic reticulum and a rather large nucleus. The latter is found in various positions and shows a conspicuous nucleolus. Numerous electron-dense granula of different shape and size and many secretory vesicles are also seen [3]. Mitochondria and Golgi bodies are scattered throughout the cytoplasm. Cell polarity is not observed. Frequently, rhogocytes form small clusters within the connective tissue, but junctions with neighboring cells have not been observed. Apparently, the lamina of extracellular matrix prevents such contacts.

Various biological functions have been proposed for these cells. Clearly, the combination of enveloping lamina and slit apparatus might act as a molecular sieve [1]. However, neither the direction nor the particles of this hypothetical ultrafiltration are known. The enveloping lamina of rhogocytes is in direct contact with the hemolymph, but for a possible uptake of hemolymph proteins by rhogocytes, experimental evidence is lacking. However, rhogocytes play a proven role in metal ion homeostasis [8], are involved in transport and storage of nutrients [1], [3], [9], [10], and act in detoxification [11] and defense systems [12]. Additionally, they participate in calcium mobilization for shell formation [13].

Since gastropod rhogocytes often contain, in lacunae of the endoplasmic reticulum, large amounts of the extracellular oxygen carrier hemocyanin, these cells have been addressed as the site of hemocyanin biosynthesis [14]–[17]. Hemocyanin is a multimeric blue copper protein and structurally known in detail (for review, see [18]). Nevertheless, in these papers it could not be excluded that instead of (or in addition to) synthesizing hemocyanin, the rhogocytes absorb hemocyanin from the hemolymph for degradation. Ultimately, their role in hemocyanin synthesis has been confirmed in the vetigastropods *Haliotis tuberculata* and *Megathura crenulata* by *in situ* hybridization [2], [19].

Members of the freshwater snail family Planorbidae, for example *Planorbarius corneus* and *Biomphalaria glabrata*, express only minute amounts of hemocyanin [20]; instead, they use a multimeric hemoglobin for oxygen transport; this protein has been studied structurally and functionally (*e.g.* [20]–[22]). The site of hemoglobin biosynthesis in red-blooded snails is unknown, although based on ultrastructural and histochemical results, the rhogocytes have been proposed as candidates [23]. *B. glabrata* is an intensively studied species because of its medical relevance as intermediate host of the trematode parasite *Schistosoma mansoni* that causes the severe tropical disease bilharziosis. Moreover, a genome project on *B. glabrata* exists [24], and we have studied hemoglobin and other hemolymph proteins from this snail [20], [25]. Therefore, for the present study on a snail expressing hemoglobin, we chose *B. glabrata*.

Here we present the ultrastructure of rhogocytes in more detail than previously, and for the first time in 3D, with focus on the region of the slit apparatus. Moreover we studied the role of rhogocytes in the biosynthesis of respiratory proteins and provide evidence as to how these proteins are secreted into the hemolymph. In addition we have collected data regarding other biological functions of these cells.

## Results

### Tissue Distribution and Abundance of Rhogocytes

**Figure 1:**
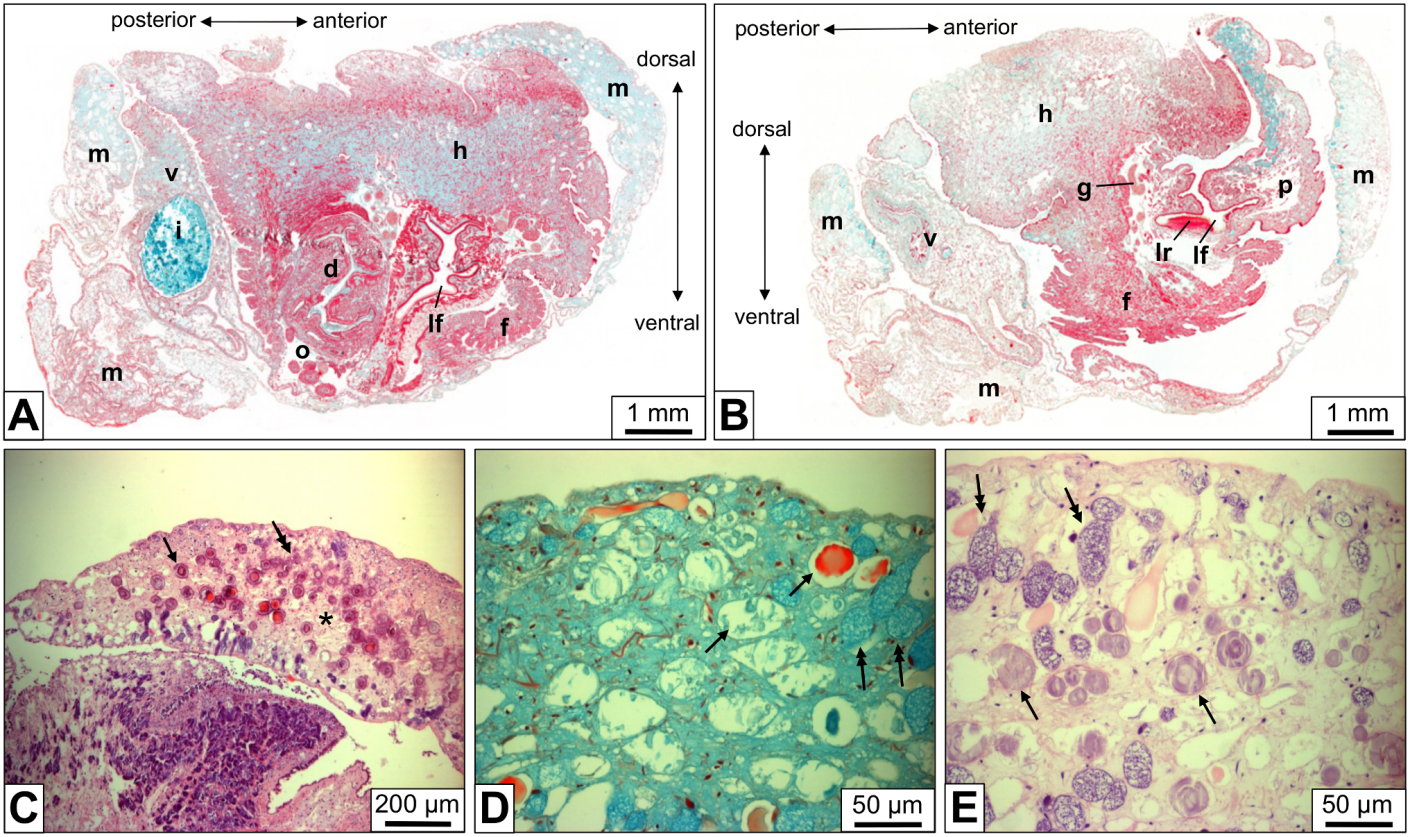
Light microscopy of paraffin-embedded tissue sections of *B. glabrata*. (**A**) Total scan of a diagonal cut through the animal. (**B**) Total scan as in (A), but from a section 0.4 mm closer towards the body surface. d, dart sac; f, foot; g, ganglion; h, head; i, intestine with food mass; lf, lime fold; lr, lime ridge; m, mantle; o, ovo-testis; p, propodium; v, visceral hump. (**C**) Mantle tissue section, showing many scattered cells (arrow, double arrow) embedded in the connective tissue between muscle cells (asterisk). (**D**, **E**) At higher magnification, weakly stained cells filled with a homogeneous material (arrows) are discernable from somewhat smaller, strongly stained cells that are filled with a dense lamellar material (double arrows). Corresponding electron microscopical images (see Fig. 3) revealed that the strongly stained cells are rhogocytes and that the lamellar material is mostly endoplasmic reticulum and dense granula. The weakly stained cells are mucus glands. Movat’s pentachrome staining (A, B, D) and hematoxylin & eosin staining (C, E) were applied.

A single *B. glabrata* individual was entirely cut into sections of 3–5 µm thickness. Consecutive stained sections were analyzed by light microscopy (Fig. 1A, B). We found the foot densely packed with horizontally and vertically oriented muscle cells. The mantle contained muscle cells and in addition secretory cells with material that might be used for shell formation. Moreover, the connective tissue of both body parts showed, besides mucus glands, many solitary rhogocytes and small rhogocyte clusters (Fig. 1C). Rhogocytes are readily detected in the light microscope by their strong staining and lamellar substructure (Fig. 1D, E); the latter is most probably due to their rich endoplasmic reticulum. Electron microscopy of comparable tissue sections (see below) was required to establish this correspondence. Since the rhogocytes were more abundant in mantle than in foot, we used primarily mantle tissue in our subsequent study.

### Tracing Hemoglobin by Immunohistochemistry and *in situ* Hybridization

**Figure 2:**
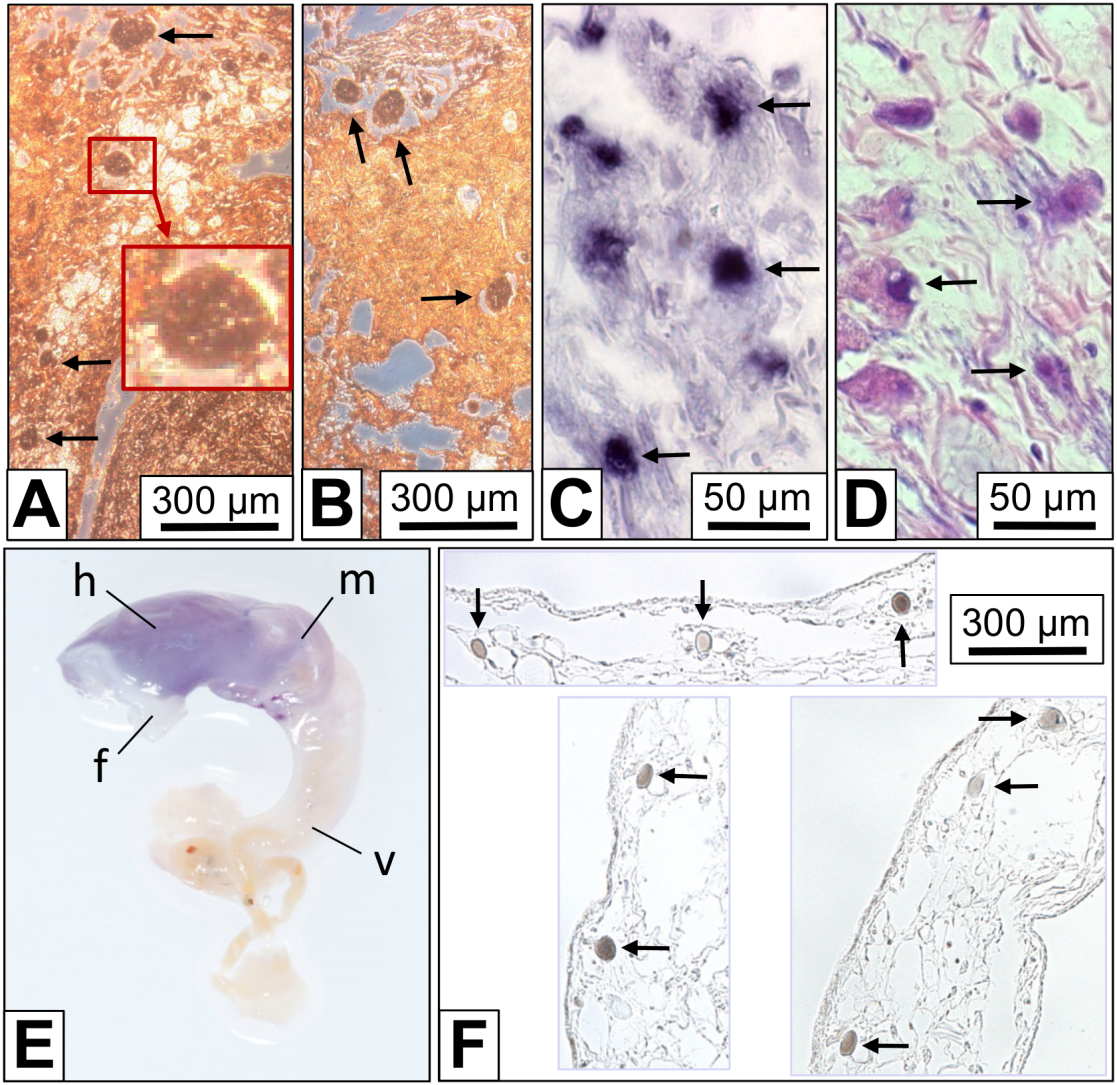
Detection of *B. glabrata* hemoglobin (BgHb) in tissue sections and whole mounts. (**A**, **B**) Indirect immunohistochemistry with rabbit anti-BgHb antibodies on mantle tissue sections. The background staining might result from hemoglobin freely dissolved in the hemolymph spaces. The strongly stained cells (arrows) are morphologically identified as rhogocytes. The insert shows an enlargement of the region in the red box. (**C**) *In situ* hybridization with antisense BgHb1-h cDNA (see Table 1) performed on a mantle tissue section. Note strong reaction of cells that are morphologically identified as rhogocytes (arrows). (**D**) A section next to that seen in (C), stained with Movat’s pentachrome to visualize rhogocytes (arrows). (**E**) Whole mount *in situ* hybridization using antisense BgHb2-i cDNA (see Table 1). Note blue staining of head (→h), foot (→f) and mantle (→m), and negative reaction of the visceral hump (→v). Total length of the animal was 2 cm. (**F**) Three different paraffin-embedded tissue sections of a whole mount as shown in (E), but treated with antisense BgHb1-h cDNA. Note specific labelling of cells morphologically identified as rhogocytes (arrows).

Immunohistochemistry on *B. glabrata* tissue sections with rabbit anti-*Biomphalaria glabrata* hemoglobin (anti-BgHb) primary antibodies yielded a strong reaction of cells morphologically identified as rhogocytes (Fig. 2A, B). The weaker staining in the surrounding tissues was presumably caused by free hemoglobin in the hemolymph spaces. This suggests that in the rhogocytes a higher hemoglobin concentration than in the hemolymph exists, indicating that rhogocytes either accumulate hemoglobin from the hemolymph, or synthesize it.

To decide this question, *in situ* hybridization was performed using two digoxigenin-labeled antisense cDNA probes specific for mRNA encoding a fragment of the *B. glabrata* hemoglobin (BgHb) subunit types BgHb1 and BgHb2, respectively (Table 1). With both probes applied to paraffin-embedded mantle and foot tissue sections, we obtained a strongly positive reaction of individual cells histologically identified as rhogocytes (Fig. 2C, D). Both cDNA probes reacted in a similar pattern. By comparable experiments on whole mounts, a specific staining of head, mantle and foot was observed (Fig. 2E). Paraffin-embedded tissue sections of such whole mounts revealed a specific labelling of cells identified as rhogocytes (Fig. 2F). This confirmed rhogocytes as the site of hemoglobin biosynthesis in *B. glabrata*. It should be noted that only a fraction of the rhogocytes visible in a certain tissue reacted with the DNA probe.

**Table 1:** Antisense cDNA probes applied to *in* *situ* hybridization.

Probe for RNAencoding hemoglobinisoform BgHb1(specific for a segment of hemedomain BgHb1-h [20])	Probe for RNAencoding hemoglobinisoform BgHb2(specific for a segment ofheme domain BgHb2-i [20])
TCCGTAACCAGGTCAGGGCCA TCACCCGTGGTATCGAGTCATTTGTGAACAGTCAACAACCCCGCTGCTCTCCAGTCCAGCATTGAGAATCTGGTCGATGCTCATTTGAACTTCCAACCCAGCATTGGTCTTTCCTACTTTGGATCCGTCCAACAATACATCCATCTCTACATCGCAAGCTCTCGGTGTTGCTTCTAACAGTGATGAGGCCAAATCATGGACTAACTTGTTCGCTGCCTTCAACAAAGTCTTGAAAGAGCATTCCCTCGAGAAAATCGGTATTTCAGATAGCGATAAAAGAGCACTTGTCAGCTCCTGGAAGAAACTAACTGCTGGTGGCAGACAAAACTTCGGT	GGTGTGGCTGCCAACAGTGA TGAAGCTACATCCTGGACTAACCTTTGGGCTGCTTTCAATAAAGTTCTCAAAGAGCATTCTCTAGAAAAACTTGGAATCACTGACAATGAAAGAAAAATTCTGGTTAGTTCTTGGAAAAGATTGACAACTGAAGCCAATGGTCAGCAAAGTCTCGGAGTCAAACTAGTTCTCTGGATGTTGGACAGTGCCCAATATGCGTGACCAATTCACAAAGTTCAATGCACGCCAATCCAATGATGATTTGAAGAGAGATGCTGGCTTTCTGAAACAAGTTAAGAAAATTATTGGAGGCTTGGGCTCCTTGGTGGACGTTG

These cDNA probes specifically label mRNA encoding a hemoglobin isoform of *B. glabrata*. The primers are underlined.

### Ultrastructure of *B. glabrata* Rhogocytes

**Figure 3:**
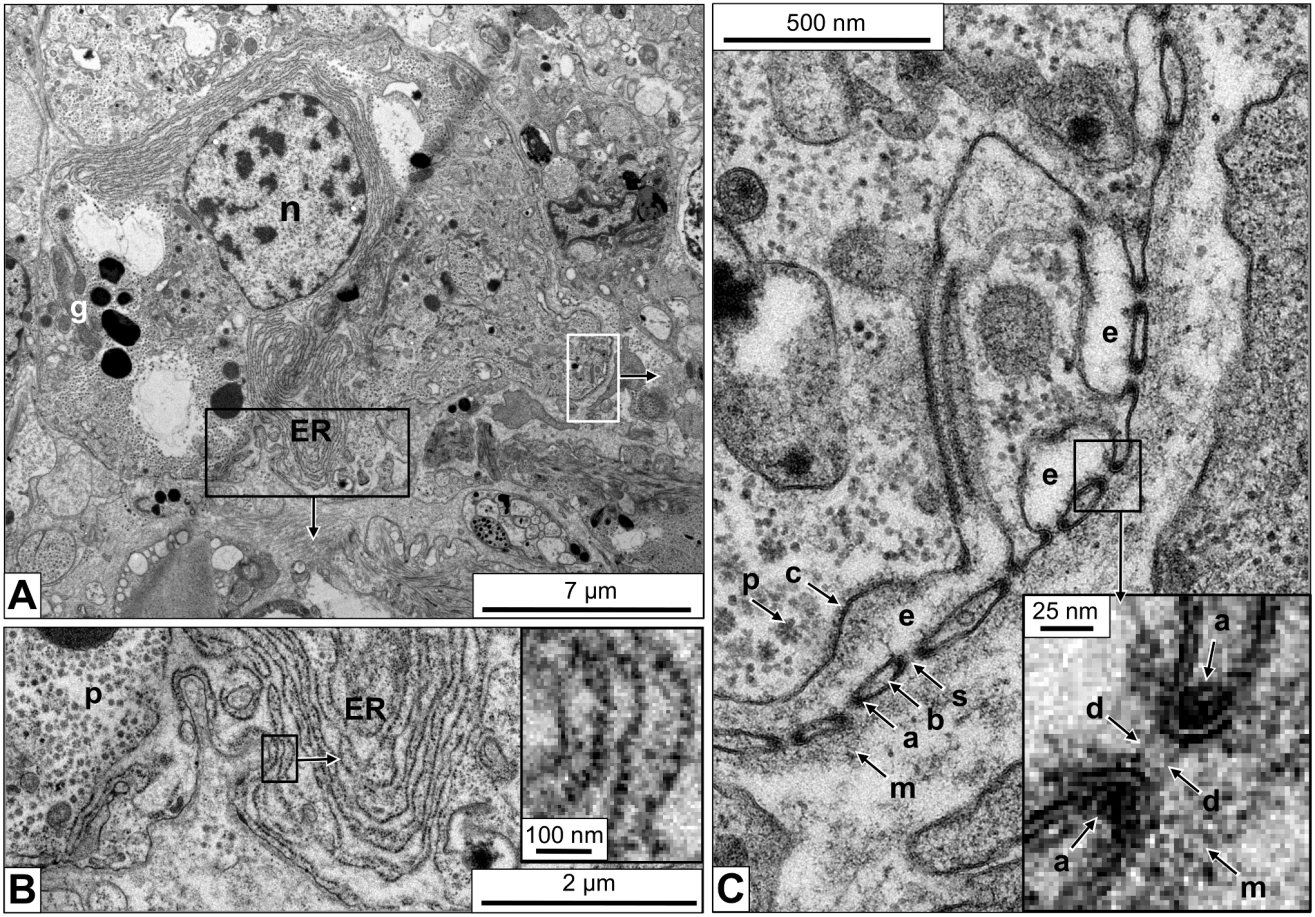
Electron microscopy of a foot tissue section showing a typical rhogocyte. (**A**) Overview of the rhogocyte. Note the large nucleus (→n), electron-dense granula (→g) and the abundant endoplasmic reticulum (→ER). (**B**) Enlargement of the region indicated by a black box in (A), showing that the endoplasmic reticulum (→ER) is lined with ribosomes (*i.e.* rough ER). Note in the adjacent ER lacuna the flocculate material that might be protein (→p). The insert in (B) shows a further enlargement of the region indicated by the black box in (B) to visualize individual ribosomes. (**C**) Enlargement of the region indicated by a white box in (A), showing several extracellular lacunae (→e) with cytoplasmatic bars (→b) and 20 nm slits (→s). In the bars, adjacent to the slits, electron-dense material is visible that was later shown to contain actin (→a). Also note the lamina of extracellular matrix (→m) and the coat (→c) lining the plasma membrane at the extracellular lacunae. In the adjacent cytoplasm, the protein-like material is seen (→p). The insert in (C) shows a further enlargement of the region indicated by a black box in (C) to visualize the slit diaphragm (→d) as two parallel small rods. Also note, in this insert, the actin-containing electron-dense material (→a) and the lamina of extracellular matrix (→m). The gully grate-like cytoplasmic bars are cut here in transversal section; for a longitudinal section, see Fig. 4B.

Electron microscopy of different connective tissue sections of *B. glabrata* revealed many rhogocytes. They appear to be diverse in size and shape, but this might partially depend on their level within the section. Among the typical features of rhogocytes allowing their identification in the electron microscope are the enveloping lamina of extracellular matrix, a comparatively big nucleus, many electron-dense granula and a prominent rough endoplasmic reticulum (Fig. 3A, B). Mitochondria and Golgi bodies are also observed. The distinguishing feature of rhogocytes, pocket-like lacunae (usually 1–8 µm in diameter) created by invaginations of the plasma membrane and bridged by cytoplasmic bars, is present in many regions of the cell surface (Fig. 3C).

**Figure 4:**
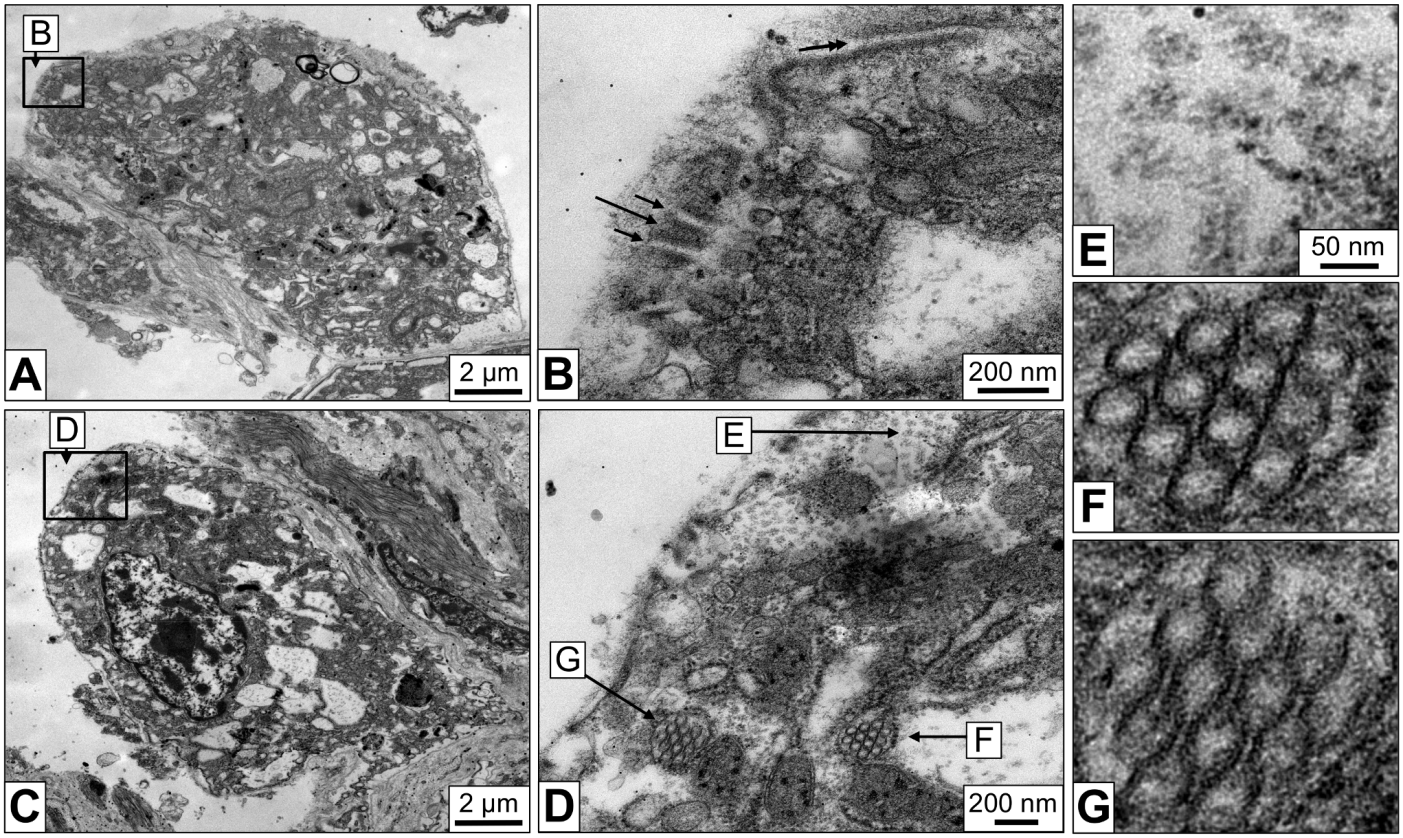
Electron microscopy of mantle tissue sections. (**A**) Rhogocyte, with the nucleus not visible in this section. (**B**) Enlargement of the area marked in (A), showing a longitudinal cut through several cytoplasmic bars (for a transversal cut, see Fig. 3C). It is obvious that the cytoplasmic bars (long arrow) border slits (short arrows) and not holes. Note that these slits can be very long (double arrow). (**C**) Rhogocyte, with the large nucleus visible. (**D**) Enlargement of the area marked in (C), showing several endoplasmic reticulum lacunae filled with circular structures *ca.* 50 nm in diameter (→F). These structures might be cylindrical hemocyanin molecules viewed from the top. However, in some areas these structures show open connections (→G) which is not explained by the typical hemocyanin structure (see, however, Fig. 10). Also note the more amorphous protein-like material in other lacunae (→E) that is interpreted as hemoglobin. (**E**–**G**) Enlargements of the regions marked in (D).

Between adjacent cytoplasmic bars a slit of *ca*. 20 nm width exists. The frequently observed cross sections through the slit apparatus show that the passage is very short (see Fig. 3C). However, the occasional horizontal sections reveal that the slits are indeed very long, but rather invariant in diameter (Fig. 4A, B). They are not straight like the slits of a gully grate, but often meander, thereby forming a labyrinth of narrow clefts at the cell surface (see also Fig. 2B in [1]). Nevertheless, the gully grate is a useful structural model for functional considerations. This model also illustrates why in electron microscopical images cross sections of the slit apparatus are much more frequent, and explains why in tissue sections many bars are not connected to the cytoplasm (see Fig. 3C): It is simply a matter of the angle and position of the ultramicrotome cut. Within the bars, accumulations of a highly dense material border the slits, and at higher magnification, cross sections reveal that neighboring bars are connected by a thin diaphragm (see insert in Fig. 3C).

The extracellular lacunae of *B. glabrata* rhogocytes can be empty, or filled with nanoscopic particles that might be protein (Fig. 4C, D). Similar particulate material is often observed in lacunae of the endoplasmic reticulum. From its amount and heterogeneous appearance we conclude that it is partially clustered hemoglobin (Fig. 4E) which agrees with a previous suggestion [23]. Occasionally, intracellular vesicles containing accumulations of electron-dense rings measuring *ca*. 50 nm across are observed (see Fig. 4D). Although this diameter is larger than the expected 35 nm, we interpret these particles as top views of hemocyanin molecule stacks (Fig. 4F). Hemocyanin is expressed in *B. glabrata* as a trace component [20]. In some views, the rings are open and connecting bridges are visible (Fig. 4G). We think that this somehow results from the plane of section through the stacks of hollow cylinders, but we admit that this explanation is rather speculative (see also below).

**Figure 5:**
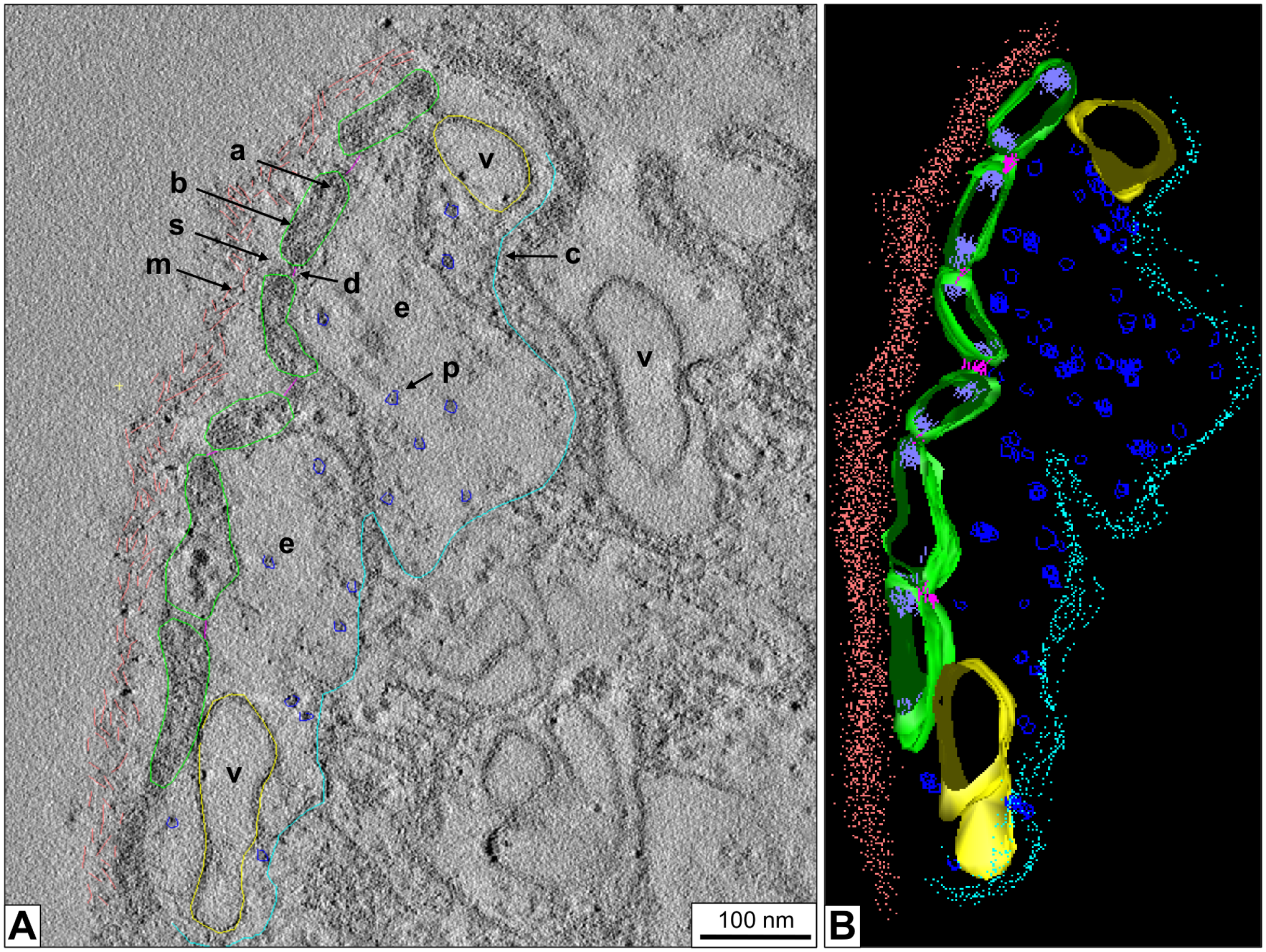
3D ultrastructure of a rhogocyte region with slit apparatus. (**A**) Electron tomogram slice of chemically fixed mantle tissue, superimposed by a 3D reconstruction performed in IMOD. (**B**) The corresponding 3D model visualized and segmented in IMOD. Note the enveloping lamina of extracellular matrix (→m, salmon), the extracellular lacuna (→e) filled with protein-like particles (→p, blue), the coated plasma membrane (→c, cyan), the bridging cytoplasmic bars (→b, green), the slits (→s) with the diaphragm (→d, magenta). Vesicles (→v, yellow) inside the extracellular lacuna and the neighboring cytoplasm are also seen. Highly dense material (→a, purple) is observed at the cytoplasmic bars adjacent to the slits; it might contain actin bundles (see Fig. 7).

**Figure 6:**
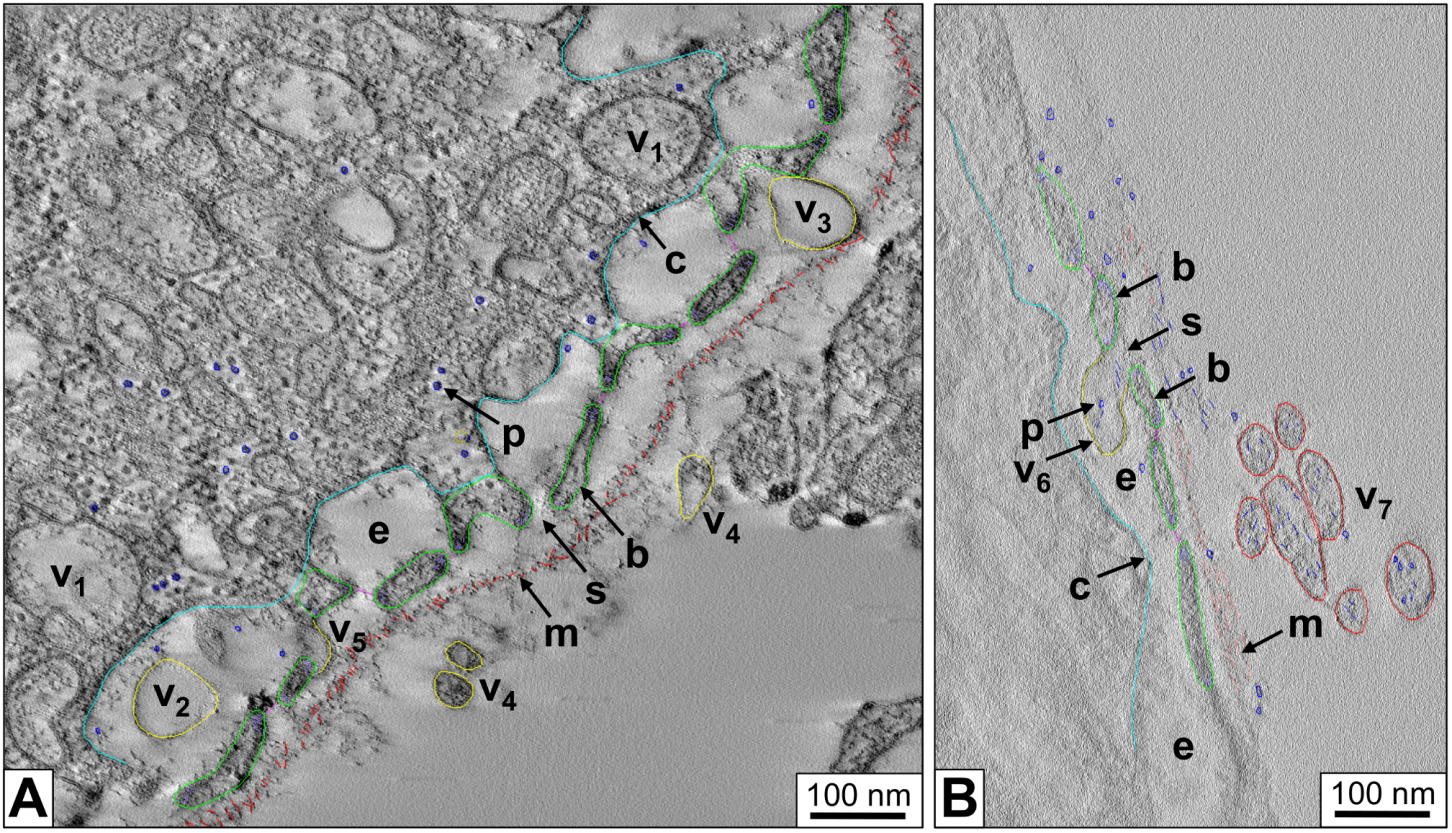
3D ultrastructure of a rhogocyte periphery with different locations of vesicles. Electron tomograms with superimposed 3D reconstructions are shown. (**A**) Region showing the coated plasma membrane (→c, cyan), several extracellular lacunae (→e), diaphragmatic slits (→s) and the lamina of extracellular matrix (→m, salmon). Note vesicles (yellow) present in the cytoplasm (→v_1_), the extracellular lacunae (→v_2_), between slit apparatus and lamina (→v_3_), and outside of the latter in the adjacent hemolymph (→v_4_). A vesicle probably fused with the slit apparatus is also seen (→v_5_). (**B**) Region showing, in an extracellular lacuna (→e), a vesicle (→v_6_, yellow) in open contact with a diaphragmatic slit (→s) formed by neighboring cytoplasmic bars (→b, green). The vesicle contains protein-like material (→p, blue) that is likely to be transported through the slit. Note similar vesicles (→v_7_, red) outside of the lamina of extracellular matrix (→m).

Electron tomographic 3D reconstructions of *B. glabrata* rhogocytes show more details of the slit apparatus. They suggest that the diaphragm is indeed a bundle of filaments. Moreover, in the extracellular lacunae globular particles *ca*. 25 nm in diameter and vesicles are observed (Fig. 5). Occasionally, vesicles are fused with the plasma membrane which brings their content in open contact with the extracellular lacuna of the slit apparatus; intracellular vesicles (frequently with a visible coat) are often nearby. Moreover, uncoated vesicles are found between lamina and slit apparatus; on some occasions they are directly fused with a slit, with their interior openly contacting the extracellular lacuna (Fig. 6A). Uncoated vesicles within the lacunae in open contact with a slit, and similar vesicles outside of the enveloping lamina are also present (Fig. 6B).

### Immunogold Localization of Actin

**Figure 7:**
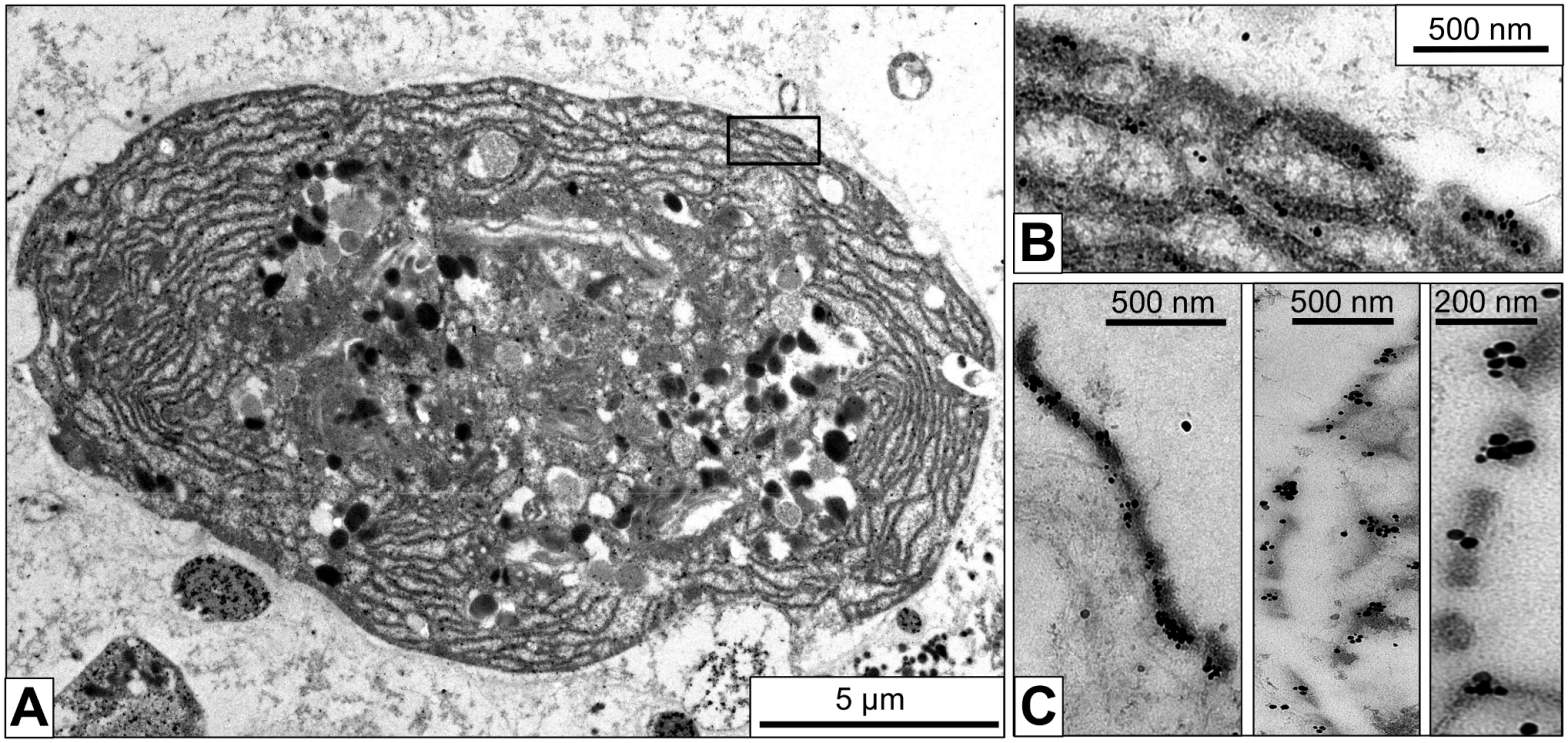
Immunogold localization of actin in the slit apparatus. (**A**) Electron microscopy of a rhogocyte in a mantle tissue section labeled with immunogold particles against actin. (**B**) The region marked by a box in (A) imaged at higher magnification, showing gold labeling. (**C**) Part of the slit apparatus of three different rhogocytes, showing anti-actin immunogold labeling in the electron-dense regions of the cytoplasmic bars adjacent to the slits.

Actin is a key player in regulating the trafficking through the slits in mammalian podocytes and insect nephrocytes [6], [26]. Therefore, immunogold electron microscopy using a monoclonal anti-actin primary antibody was performed. This antibody specifically recognizes all known actins throughout the animal kingdom and beyond [27]. Gold particles were detected in the cytoplasm and the nucleus. This was expected as actin is a component of the cytoskeleton and chromatin complexes. In addition, strong labeling of the cytoplasmic bars of the slit apparatus by gold particles was observed, notably in the regions of the dense material (Fig. 7). Consequently, a major component of this material is likely to be actin.

### Detection, Structure and Localization of Nephrin

**Figure 8:**
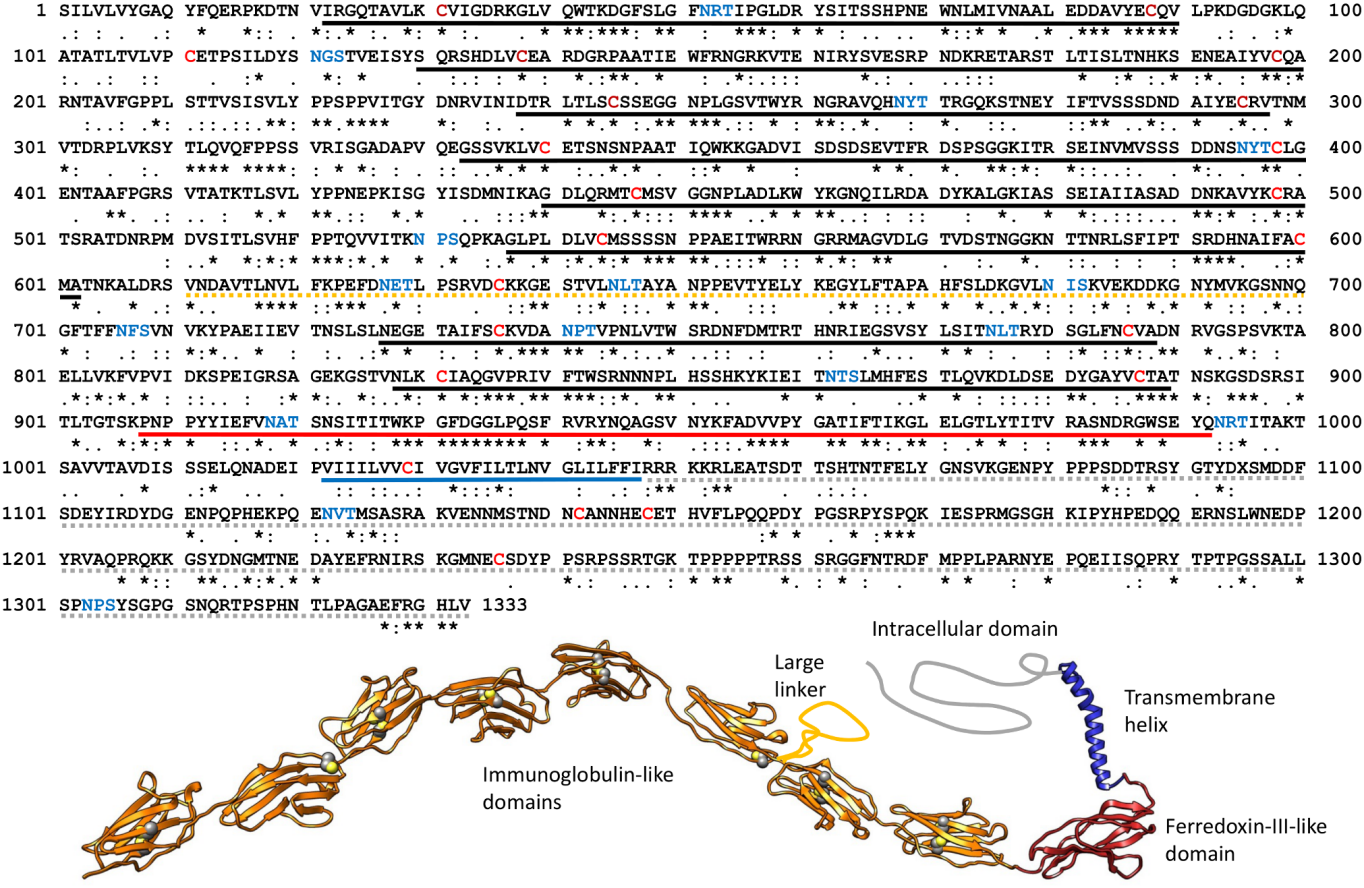
Primary structure of *B. glabrata* nephrin. Its sequence relationships to human nephrin (AF190637.1) as deduced from a pair-wise alignment are shown by symbols for identical (*), isofunctional (:) and similar (.) amino acids. Note that gaps in the alignment are not considered here. The predicted structural domains as defined by Kestilä *et al*. [28] are indicated by thick lines (black, extracellular immunoglobulin-like domains; orange dotted, large linker; red, extracellular ferredoxin-III-like domain; blue, transmembrane helix; grey dotted, intracellular domain). Also indicated are the cysteines (red letters), most of which form a disulfide bridge within the immunoglobulin-like domains. Moreover, the potential attachment sites for N-linked glycans are highlighted (blue letters). The sequence is available under GenBank accession number KJ829367. Also shown is a predicted 3D structure as obtained by homology modelling; regions with no template available are sketched. (The same color code as above, except for the immunoglobulin-like domains.) Cysteines forming disulfide bridges are highlighted.

The phylogenetic relationship between mammalian podocytes and insect nephrocytes has been demonstrated by showing that related proteins, notably nephrin, form the slit diaphragm [7]. To prove whether this is also the case with the molluscan rhogocytes, we screened the *B. glabrata* genome database and retrieved a gene encoding a complete nephrin; the predicted amino acid sequence encompassed 1333 amino acids (Fig. 8) plus N-terminally the fragment HYFV of a signal peptide. Transcriptome data confirmed this sequence. In a pair-wise alignment it showed 26% identity and 42% similarity with human nephrin; note the corresponding symbols in Fig. 8. Its head and central part (1112 amino acids aligned) shared 63% identity with a nephrin fragment retrieved from the genome data of the sea hare *Aplysia californica* (XM_005106803), whereas its tail (670 amino acids aligned) was 40% identical with a nephrin tail fragment of the bivalve *Crassostrea gigas* (EKC26159). For all the structural domains present in human nephrin [28], an equivalent sequence in *B. glabrata* nephrin exists. Using a variety of crystal structures as templates, we produced a preliminary homology model of the protein (see Fig. 8).

**Figure 9:**
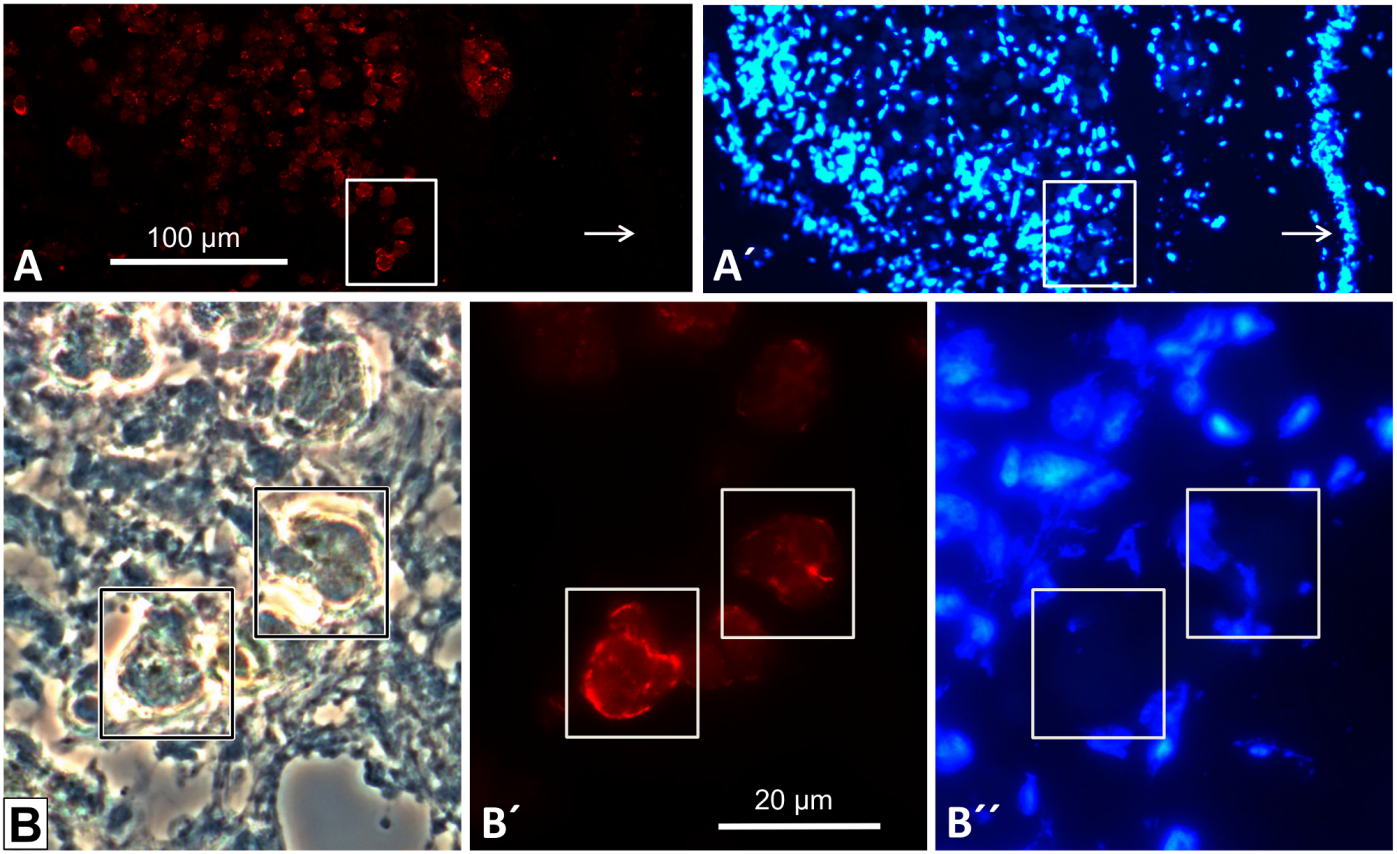
Immunofluorescence microscopy of a mantle frozen tissue section with anti-nephrin antibodies. (**A**) Cluster of rhogocytes at 250x primary magnification, showing positive immune reaction with guinea pig anti-nephrin antibodies. Note negative reaction of the mantle epithelium (arrow). (**A’**) The same section as in (A), labeled with the DNA stain DAPI (diamidino-2-phenylindole) to visualize the cell nuclei (arrow, mantle epithelium). (**B–B”**) The boxed area in (A, A’) at 1000x primary magnification, shown in phase contrast optics (B), epifluorescence optics with anti-nephrin antibodies (B’), and epifluorescence optics with DAPI stain (B”). Two rhogocytes are highlighted in boxes. Note that the positive reaction is mostly restricted to the cell periphery.

Since nephrin deficiencies are the cause of several kidney diseases [28], various polyclonal anti-nephrin antibodies are commercially available that are directed against the intracellular domain. Immunofluorescence microscopy of *B. glabrata* tissues with guinea pig anti-nephrin antibodies revealed strong labeling of the periphery of cells morphologically identified as rhogocytes (Fig. 9). This is evidence that nephrin is a component of the slit apparatus.

### Responses of Rhogocytes to Deprivation of Food and Cadmium Stress

**Figure 10:**
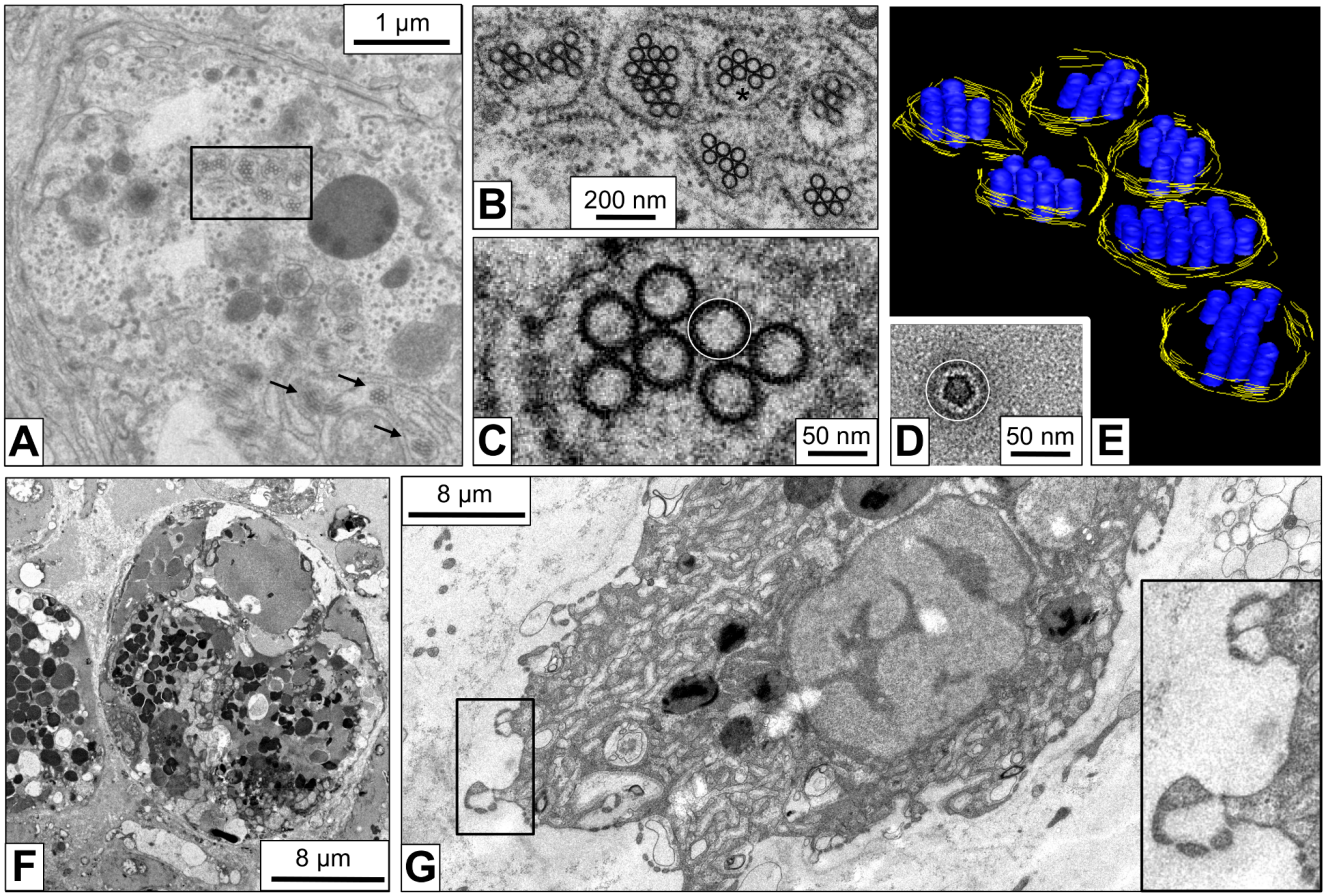
Responses of rhogocytes to deprivation of food and cadmium stress. (**A**) Electron microscopy of a rhogocyte from a snail deprived of food for 96 hours. Electron-dense granula and extracellular lacunae with slit apparatus are rarely seen, but vesicles containing hemocyanin-like particles are abundant (box and arrows). (**B**) Higher magnification of the region marked in (A) by a box. (**C**) Enlargement of the region marked in (B) by an asterisk. Note that the 50 nm rings show no connections, in contrast to those in Fig. 4G. (**D**) *B. glabrata* hemocyanin molecule extracted from an electron microscopical image of negatively stained hemolymph proteins (for details, see [20]). Note that its outer diameter (*ca.* 35 nm) corresponds to the diameter of the internal material of the 50 nm rings. In other words, the black annulus visible in (C) is heavy metal stain and not the protein cylinder wall, and corresponds to the dark uranyl acetate halo surrounding the molecule in (D). The thin white circle in (C, D) indicates the dimension of 50 nm. (**E**) 3D model derived from electron tomography of the area shown in (B). Note that the hemocyanin-like particles now appear as stacks of short cylinders. This is compatible with the quaternary structure of *B. glabrata* hemocyanin which is a hollow cylinder 35 nm in diameter and 18 nm in height (see [18]). (**F**) Rhogocyte of a cadmium-contaminated animal (0.05 mg/l CdCl_2_, 48 h). Note the large number of electron-dense granula (for comparison, see Fig. 3; for quantification, see Table 2). (**G**) Rhogocyte of a cadmium-contaminated animal (0.05 mg/l CdCl_2_, 96 h), suggesting significant increase of the filtrating cell surface. The insert shows an enlargement of the boxed area.

Tissues from *B. glabrata* individuals that had starved for 96 hours prior to fixation were studied in the electron microscope. In comparison to the controls, the rhogocytes seemed to show a decrease in plasma membrane invaginations and electron-dense granula (Fig. 10A), although quantified data are not available as yet. The major change observed was the presence of a larger number of the small vesicles filled with ring-like particles 50 nm in diameter (Fig. 10B, C). Such vesicles have also been observed in untreated animals, but less frequent (see Fig. 4), as deduced from visual inspection of a limited number of cells. (In view of their sporadical appearance, we did not try to quantify these tiny vesicles.) As mentioned above, we interprete the 50 nm structures as hemocyanin. Gastropod hemocyanin is usually a cylindrical didecamer 35 nm in diameter and 35 nm in hight, with an outer wall and an internal collar (for review, see [18]). The hemolymph of *B. glabrata* contains, besides large amounts of hemoglobin and a minor portion of a dodecahedral acetylcholine-binding protein, traces of a “truncated” hemocyanin. It is a solitary decamer (35×18 nm) exclusively composed of a cylinder wall, with the internal collar lacking [18], [20], [25]. The top-view of such a decamer obtained from the hemolymph is shown in Fig. 10D. The apparent discrepancy of the cellular rings in tissue sections and negatively contrasted hemocyanin molecules in diameter (50 versus 35 nm) is explained as a staining artifact (see Fig. 10B–D).

The 3D reconstruction of a group of such vesicles revealed that the rings represent the top-view of elongated stacks of short cylinders (Fig. 10E), and therefore might indeed represent stacks of hemocyanin decamers. Investigation of hemolymph proteins extracted from the starved animals prior to their fixation yielded a significant enrichment in hemocyanin decamers (not shown). Although this has to be verified by other methods, it fits the hypothesis that in starved snails the hemocyanin biosynthesis is up-regulated. In contrast, the possible idea that under such conditions the rhogocytes accumulate hemocyanin from the hemolymph for catabolism is not supported.

Animals that were normally fed for 12, 48 and 96 hours in tap water containing 0.05 or 0.1 mg/l cadmium chloride showed many but considerably small rhogocytes, indicating that their number has increased by cell division. Compared to the control animals, many of these rhogocytes carry significantly more electron-dense granula, as deduced from visual inspection of electron micrographs (Fig. 10F). Using the EMAN2 software [29], this increase could be quantified and thereby confirmed (Table 2). Moreover, it appeared that such rhogocytes contain more mitochondria, although in this case we lack quantified data. The effects of the cadmium contamination were obvious already after 12 hours of exposure and more intense after 48 and 96 hours. There was no significant difference detected between 0.05 and 0.1 mg/l CdCl_2_. An increase of hemocyanin-containing vesicles was not observed, unlike in rhogocytes from starved animals. However, extracellular lacunae with slit apparatus were much more frequently detected in these cells, often due to intensive folding of the cell surface, suggesting an overall increase of the filtration capacity (Fig. 10G).

**Table 2:** Number of electron-dense granula in individual rhogocytes from untreated and CdCl_2_-contaminated animals.

untreated			contaminated		
2	4	6	8	14	21
3	5	6	8	14	22
3	5	8	10	16	24
3	5	8	10	16	24
3	5	9	12	20	28
3	5	9	13	21	
2	5	9	13	21	
Ø = 5.2			Ø = 15.9		

21, respectively 19 electron micrographs, each showing a single rhogocyte, were analyzed by using the module “e2boxer” of the EMAN2 software package [29] in a semi-automated mode.

## Discussion

From their prominent rough endoplasmic reticulum, rhogocytes have been addressed as hemocyanin producing cells four decades ago by Sminia [3]. Sminia & Vlugt-van Daalen [16] showed images of *Helix aspersa* rhogocytes with numerous vacuoles derived from endoplasmic reticulum and apparently filled with hemocyanin molecules. However, it remained open whether this hemocyanin was just synthesized and about to be secreted, or taken up from the hemolymph and about to be degraded. Much later, it was demonstrated by *in situ* hybridization in the marine gastropods *Haliotis tuberculata* and *Megathura crenulata* that the rhogocytes do indeed synthesize the hemocyanin [2], [19]. In the present study, by *in situ* hybridization we have confirmed the early hypothesis of Sminia *et al*. [23] that in planorbid snails the hemoglobin is also synthesized by rhogocytes. They produce both hemoglobin polypeptides, but it remains open whether this happens together in a single cell. We observed that not all rhogocytes in the respective tissue sections were labeled. Probably, not all rhogocytes produce this protein at the same time, or the encoding mRNA, being a transient product, was degraded in some rhogocytes at the time of the experiment.

The capability of rhogocytes to synthesize both hemocyanin and hemoglobin is interesting with respect to their protein structure. The two respiratory proteins are completely unrelated phylogenetically, but in gastropods they share a quite unusual feature: They are both composed of extraordinarily large polypeptide subunits, namely 300–550 kDa in molluscan hemocyanin [18], [30] and 240 kDa in *B. glabrata* hemoglobin [20]. Within these subunits, a number of paralogous functional domains (also termed “functional units”, each with a single active site) are concatenated like a pearl chain *via* peptide linkers. Typically, their number in hemocyanin is eight, whereas 13 functional domains encompass the *B. glabrata* hemoglobin subunit. The cellular machinery for encoding, transcribing and translating such extravagant polypeptides seems to be a specific feature of rhogocytes.

The truncated hemocyanin expressed in *B. glabrata* is only a trace component in the hemolymph and is therefore certainly not required for oxygen transport [20]. If it is not just a rudiment inherited from a blue-blooded past, this protein might be expressed for the prophenoloxidase/tyrosinase activity of molluscan hemocyanin [31]. Our food deprivation experiments suggest that it is up-regulated under such stress conditions (see Fig. 10A–C), which indicates that the truncated *B. glabrata* hemocyanin is needed by the animal. In *Drosophila*, a detailed survey revealed a high complexity of down-regulated and up-regulated hemolymph proteins during starvation of larvae, and among the up-regulated proteins was a prophenoloxidase [32]. The 50 nm rings observed in *B. glabrata* rhogocytes have still to be positively identified as hemocyanin, and their apparent up-regulation has to be confirmed by quantified data. On the other hand, the truncated hemocyanin molecules of *B. glabrata* must be synthesized somewhere in rhogocytes, and the 50 nm rings are the only candidates detected as yet.

Since the respiratory proteins are synthesized in the endoplasmic reticulum, they should be secreted into the hemolymph by exocytosis *via* secretory vesicles that fuse with the plasma membrane. In this context it is most interesting to note that vesicles fused with regions of the plasma membrane that are not part of the extracellular lacunae are rarely detected. In contrast, coated vesicles filled with nanoscopic particles are frequently observed in the cytoplasm adjacent to the extracellular lacunae, and sometimes they are fused with the latter. Moreover, only the bordering of the lacunae is covered by a coat. This has also been reported for blue-blooded gastropods (e.g. [1], [2], [5]). Even if some of these vesicles are endocytotic, there should be a considerable proportion of exocytotic vesicles because there is simply no alternative secretory pathway for the respiratory proteins detected. Although coated vesicles are usually mentioned in the framework of endocytosis, exocytotic coated vesicles are not uncommon (e.g. [33]–[35]).

Often vesicles seem to reside completely in the extracellular lacunae (see Fig. 5), although it cannot be excluded that they are still in open contact with the cytoplasm. However, occasionally uncoated vesicles are localized between the enveloping lamina and the diaphragmatic slits; sometimes they seem to be fused with one of the slits, and uncoated vesicles occur also outside of the lamina (see Fig. 6). This is not compatible with endocytosis, but an additional argument for exocytotic activity. It could well be that some of these vesicles are exo-endocytotic and follow something that has been termed “kiss and run” mechanism [36]. They might temporarily fuse with the plasma membrane and release most of their protein cargo into the extracellular lacuna. Then, according to the “kiss and run” hypothesis, the reverse process takes place in that the same vesicle undergoes endocytosis. Thereby it collects, probably besides a portion of the just released protein, other substances present in the extracellular lacuna which are then further processed in the cell. The endocytosis of exocytotic vesicles would also maintain membrane integrity and balance. Processes like this have been studied in detail in other cell types, for example in synaptic vesicle exocytosis (for review, see [37]).

Small particles in the hemolymph, for example heavy metal ions such as cadmium, should freely pass the slit diaphragm. The driving force might either be muscle contraction or diffusion. The response of the *B. glabrata* rhogocytes to cadmium contamination appears to be cell division and formation of more extracellular lacunae with diaphragmatic slits, both resulting in a significant increase of the overall filtration surface. Within the rhogocytes of contaminated animals, the number of dense granula was significantly increased (see Fig. 10F). This reflects the role of these granula in heavy metal accumulation [10], [11], [38].

In the “kiss and run” model, the extracellular lacunae would be periodically or constantly enriched with newly synthesized respiratory proteins which would then follow the diffusion gradient outwards into the hemolymph**–**provided they are able to pass the diaphragmatic slits. The sieve structure of rhogocytes (“pore cells”) has been analyzed, in *Lymnaea stagnalis*, by Boer & Sminia [5]. They showed that the diaphragm between adjacent cytoplasmic bars (or “tongues”) has a tooth-like structure, with the “teeth” from the opposite sides of the slit contacting each other *via* a bridging material. Teeth of the same row are *ca*. 20 nm apart, and the slit width is also *ca*. 20 nm. This means that the diaphragm has holes of 20×20 nm, which is much larger than the 4×14 nm holes reported for the podocyte diaphragm (see [39], and literature cited therein). On the other hand, the podocyte slit diaphragm has to prevent the passage of serum albumin (about 8×8×3 nm), whereas the gastropod hemolymph proteins are larger than 20 nm. Indeed, Boer and Sminia [5] demonstrated by using gold particles that only sizes below 20 nm were ingested by the rhogocytes.

This would mean that under normal conditions, the 35×35 nm hemocyanin didecamers of *L. stagnalis* are excluded from passage. Provided that in *B. glabrata* the diaphragm architecture is similar, the 22–25 nm hemoglobin particles would also have difficulties to pass, and even more so the 35×18 nm hemocyanin decamers. Also, the dodecahedral 25 nm acetylcholine-binding protein of *B. glabrata* [25] would be excluded. We think that it is not by chance that the holes of the molecular sieve are slightly smaller than the multimeric hemolymph proteins. Indeed, the sieve has exactly the right size to block their inward migration, without providing a diffusion barrier for smaller particles.

If the slit diaphragm is indeed a molecular sieve for size-selective inward passage of hemolymph components, which is also deduced from its similarity to the diaphragm in podocytes and nephrocytes [1], [14], the outward passage of the freshly synthesized, and exocytosed, hemoglobin and/or hemocyanin requires a gating mechanism. We speculate that a chemical signal released during exocytosis stimulates the actin bundles at either side of the diaphragmatic slits to interact with motor proteins which temporarily enlarges the holes of the sieve. On such occasions, it might even be possible that entire vesicles pass through which would explain situations as shown in Fig. 6. Likewise, Watiovaara *et al*. [39] proposed that the podocyte slit diaphragm might dynamically change in dimensions, pore size and appearance.

This idea stimulated us to have a closer look on the biochemistry of the rhogocyte slit diaphragm. The major protein constituting the diaphragm in podocytes and nephrocytes is nephrin (for review, see [40]). This member of the immunoglobulin superfamily belongs to the cell adhesion proteins (Ig superfamily type C2; see [41]). It is also involved in signaling in that it transduces, together with neph1, a signal-induced actin polymerization [42]. The sequence and homology model of *B. glabrata* nephrin presented here (see Fig. 8) shows that all important features known from human nephrin are conserved (for the latter, see [28]): the eight immunoglobulin-like domains stabilized by a disulfide bridge, the exceptionally long linker between domain 6 and domain 7, the fibonectin-III-like domain, the single α-helix representing the membrane anchor, the tyrosine- and serine-rich intracellular domain that opens possibilities for phosphorylation, and a variety of attachment sites for N-linked glycans (see Fig. 8). In human nephrin, these glycans play a role in the plasma membrane localization of nephrin [43]. In the course of our study we additionally retrieved a comparable nephrin sequence from the genome database of the sea hare *Aplysia californica* (XM_005106803) which indicates that nephrin is a rhogocyte component in blue-blooded gastropods as well (for *Aplysia* hemocyanin, see [44]). In the “tooth model” of the diaphragm developed by Boer & Sminia [5], nephrin should be a major component of the “teeth”. The latter have a length of 10 nm to bridge the 20 nm slit from opposite sides. In our homology model of nephrin (see Fig. 8), the total length of the nine extracellular domains is *ca.* 35 nm. This would be more than enough to allow a certain stretching of the teeth (see above), in order to enlarge the holes of the sieve.

Morphological similarities summarized by Haszprunar [1], notably the enveloping lamina, the underlying extracellular cisternae, the cytoplasmic bars, the diaphragmatic slits, and the fenestration of the diaphragms suggest a common phylogenetic and developmental origin of rhogocytes, podocytes and nephrocytes [1], [6]. This is now further supported by our immunogold localization of actin in the highly dense material at the edges of the cytoplasmic bars forming the slits (see Fig. 7). In podocytes and nephrocytes, actin in combination with motor proteins plays an important role in maintaining the architecture and filtration function of the slit apparatus [6], [26]. The molecular structure of the slit diaphragm of podocytes and nephrocytes has been studied in detail and revealed an intricate network of two proteins, nephrin and neph1 (termed Sns and Kirre in *Drosophila*), that together form the molecular sieve [6], [7], [28]. These results confirmed the homology of podocytes and nephrocytes [7]. Our discovery and localization of actin and nephrin in *B. glabrata* is an additional molecular argument in favour of the largely ultrastructure-based hypothesis that molluscan rhogocytes, arthropod nephrocytes and vertebrate podocytes, and probably also the phoronid cyrtocytes are closely related. This old hypothesis, summarized and substantially deepened by Haszprunar [1], links the rhogocytes phylogenetically to the metanephridial systems.

On the other hand, morphological evidence has been collected that podocytes and nephrocytes are equivalent to the terminal cell of protonephridia [45]. At a final stage of our work, Stewart et al. [46] published a paper on rhogocytes in gastropod planktotrophic larvae. Their excellent electron microscopical images document a transformation, in four stages, from protonephridial terminal cells into rhogocytes. Thus, the rhogocyte has evolutionary and developmental links to both, protonephridial and metanephridial systems. This means that their molecular sieving device, the enigmatic slit apparatus, evolved already in the late Precambrian as a basic feature of the Bilateria.

## Materials and Methods

### Ethics Statement

The aquatic snail *B. glabrata* was obtained from a freshwater tank culture (at 20°C) established more than ten years ago in our research group. All animal work has been conducted according to the national guidelines. Approval of an ethics committee was not required in case of these gastropods. The animals were fed on a daily basis on tropical fish flake food. All surgery was performed under 7% magnesium chloride/ice water anesthesia, and all efforts were made to minimize suffering.

### Sample Preparation for Electron Microscopy

1–3 mm^3^ tissue pieces extracted from *B. glabrata* mantle and foot were fixed for a minimum of 2 hours at room temperature in a freshly prepared medium of 1% glutaraldehyde in 0.02 M phosphate buffer of pH 7.2. After being rinsed in 0.1 M phosphate buffer of pH 7.2, the material was post-fixed for 1 hour in 2% osmium tetroxide in 0.05 M phosphate buffer of pH 7.2. Following washing steps in 0.1 M phosphate buffer, and dehydration in ascending concentrations of ethanol, the samples were upgraded in propylene-oxide and embedded in araldite. Ultrathin sections (70–100 nm) were cut on an ultramicrotome (Reichert Ultracut E, Leica Microsystems, Wetzlar, Germany). They were stained with 2% uranyl acetate in 50% ethanol for 10 minutes and lead citrate for 2 minutes [47], [48]. On sections that would be used for collecting tilt series, one drop of 10 nm gold particles (1∶50 diluted with water) was applied for 5 minutes on each side of the grid.

For immunoelectron microscopy, 1–3 mm^3^ tissue pieces extracted from mantle and foot were fixed for 1.5 hours in a solution of 3% paraformaldehyde and 0.1% glutaraldehyde in 0.1 M phosphate buffer pH 7.3. After washing steps in 0.1 M phosphate buffer and dehydration in ascending concentrations of ethanol, the material was embedded in LR-White and gelatin caps and polymerized in UV-light at 4°C for 3 days, as previously described [49]. A monoclonal anti-actin antibody termed “clone C4” (from Seven Hills Bioreagents, Cincinnati, USA) was applied to ultrathin sections of mantle and foot tissue. This antibody was initially characterized and successfully used in immunoelectron microscopy by Lessard [27]; according to the data sheet it is directed against actin throughout the animal kingdom. Nanogold-labeling was silver-enhanced according to Danscher [50]. The sections were contrasted with 2% uranyl acetate and lead citrate [47], [48].

Some *B. glabrata* individuals were deprived of food and maintained for 96 hours in tap water. Other individuals that were fed normally were exposed to cadmium containing conditions (CdCl_2_). Tap water was used as exposure medium. The cadmium concentrations for exposure were not effectively measured, but assigned nominally. Different concentrations of cadmium chloride (0.05 mg/l and 0.1 mg/l) and different durations of the treatment (12, 48 and 96 hours) were tested. The fixation of mantle and foot tissues followed as described above.

The extraction of hemolymph proteins from snails was done as described [20], [22]. For negative staining of proteins on carbon-coated copper grids, the droplet method was applied with 1% uranyl acetate [51], [52].

### Electron Microscopy

Electron micrographs were taken by a TemCam-F416 4K 4K CCD camera (TVIPS, Gauting, Germany) operated on a FEI Tecnai12 transmission electron microscope at 120 kV. In order to view the cells in 3D [53], [54], tilt series over a tilt range of −60° to +60° with 1.5° increments were recorded. The magnification of 23,000× (0.929 nm pixel size on the CCD camera) was binned by the factor 2 (2048×2048 pixels micrographs). The EM-MENU 4 software package (TVIPS, Gauting, Germany) was used to collect the tilt series.

### Image Processing and 3D Reconstruction

The set of TIFF image files was converted into a single “mrc” file using the IMAGIC software [55]. Alignments and weighted back-projection-based reconstructions of raw tilt series using approximately 15 fiducials were computed with IMOD under eTomo (which is a graphical user interface of the IMOD software package [56]). The reconstructions were further visualized using the AMIRA software package (FEI Visualization Sciences Group, Bordeaux, France & The Zuse Institute Berlin, Germany).

Counting of electron-dense granula in electron micrographs, each showing a single rhogocyte, was done by a semi-automated procedure using the module “e2boxer” implemented in the EMAN2 software package [29]. This module collects similar single particles into individual data files.

### Light Microscopy

Whole animals were dehydrated in a series of increasing alcohol concentrations followed by xylol. Then, they were embedded in paraffin for performance of cross sections. Using a rotation microtome (Leica, Wetzlar, Germany) each investigated animal was entirely cut in subsequent sections of 3–5 µm thickness. For getting an overview, each tenth slice was stained with hematoxylin & eosin and screened by light microscopy. In specific regions of interest, two consecutive sections were stained according to published protocols [57]–[59] by Movat’s pentachrome and Azan, respectively. The third consecutive slide at such a site remained unstained for further analyses, for example *in situ* hybridization or immunohistochemistry.

Histological images were taken by using a Nikon Eclipse 80i histological microscope equipped with a Nikon DS-Fi1 digital camera; the latter was connected to a digital sight control unit (Nikon, Tokyo, Japan). Furthermore, total scans of chosen slides were generated by using the same microscope equipped with an automated scanning table (Prior Scientific, Rockland, USA). The DS-Fi1 digital camera was connected to a personal computer, and the software NIS-Elements 4.0 (Nikon, Tokyo, Japan) was applied to generate total scans. The latter encompassed a single large image of the whole histological specimen, assembled from 100–120 individual images in a 100x magnification and a resolution of 2500×1200 pixels. The whole procedure was done according to the manufacturer’s instructions and in accordance with previous publications [57]–[59].

### *In situ* Hybridization

Based on the cDNA sequences of heme domains BgHb1-h and BgHb2-i of the two *B. glabrata* hemoglobin isoforms [20], [22], specific primers for antisense cDNA probes were designed (Table 1), and generated by a commercial service (Sigma-Aldrich, Hamburg, Germany). Using these primers, isoform specific antisense cDNA probes were generated by PCR (see Table 1), and digoxigenin-labeled (Dig-labeling Kit, Roche, Mannheim, Germany).

*In situ* hybridization on whole mounts was carried out according to Streit *et al*. [60], with the following modifications: The anesthetized individuals were treated for 30 minutes with a freshly prepared solution of 0.5% KOH and 3% H_2_O_2_ to remove pigments. After the treatment with proteinase K, the animals were incubated with 4% paraformaldehyde in phospate-buffered saline (PBS). This solution was washed away with PBT (*i.e.* 0.1% Tween20 in PBS). The specimens were pre-hybridized for 3 hours, and then hybridized overnight at 42°C. They were stored at 4°C in 70% ethanol and sectioned as described above. The sections (thickness 3–5 µm) were analyzed on the Eclipse 80i histological microscope (see above).

*In situ* hybridization on paraffin-embedded tissue sections was done according to Albrecht *et al.* [2], with the following modifications: The hybridization (10 ng/µl) took place overnight at room temperature. The sections were rinsed with 2x saline-sodium citrate (SSC) buffer and treated for 10 minutes with Tris/BSA (*i.e.* 100 mM Tris/HCl, 150 mM NaCl, 0.1% bovine serum albumin (BSA), pH 7.5). The sections were further incubated with anti-digoxigenin-AP Fab fragment (diluted 1∶5000 in Tris/BSA) at room temperature for 1 hour. Staining reactions were done using nitroblue tetrazolium chloride (NBT) and 5-bromo-4-chloro-3-indolylphosphate (BCIP) in 100% dimethylformamide.

### Immunohistochemistry and Immunofluorescence Microscopy

Immunohistochemistry on paraffin-embedded tissue sections was performed according to the MaxLSABTM Rabbit HRP Detection Kit (Max Vision Biosciences, Washington, USA). Rabbit antibodies specifically directed against *B. glabrata* hemoglobin [20], [22] were diluted 1∶100,000 with 50 mM Tris/HCl, pH 7.6. Biotinylated anti-rabbit secondary antibodies included in the kit were used. The tissue sections were incubated with the primary antibody for 1 hour at room temperature and rinsed with 50 mM Tris/HCl, pH 7.6. The color reaction was stopped after 10 minutes and the sections were air dried and mounted.

Immunofluorescence microscopy was done on 7 µm thick cryo sections (cryotome HM 500 OM, Microm, Walldorf, Germany) according to the method described by Schaffeld & Markl [61]. Guinea pig polyclonal anti-nephrin antibodies NPHN/NPHSI from Acris Antibodies GmbH (Herford, Germany) were applied at a dilution of 1∶50. Images were recorded on a Leitz DM RBD fluorescence microscope (Leica, Wetzlar, Germany).

### Sequence Databank Mining and Homology Modelling

The genomic databank of *B. glabrata*, available at vectorbase.org, was screened for nephrin-like sequences using the human nephrin cDNA (AF190637.1) as probe. The positives provided LGUN_random_Scaffold858. The latter was further analyzed using Genewise (http://www.ebi.ac.uk/Tools/psa/genewise/) for comparing it to a deduced nephrin-like sequence from the gastropod *Aplysia californica* (XM_005106803) and a nephrin-like sequence fragment from the bivalve *Crassostrea gigas* (EKC26159). The resulting intron/exon structure was manually optimized and verified by mapping BB02 strain predicted transcripts (vectorbase.com). The resulting longest open reading frame codes for 1337 amino acids. The deduced primary structure was cross-checked by performing blast analyses resulting in positive hits covering 50% of the sequences in mean and providing e-values ranging from 4e^−131^ to 0 within the best 100 hits. The sequence is available under GenBank accession number KJ829367. Homology modelling and visualization of the 3D model of nephrin was done as described for other proteins [25].
